# Resistance Training with Aerobic Active Recovery in Obesity and Metabolic Syndrome: Translational Rationale, Proposed Training Protocol, and Multi-Omics Research Framework—A Narrative Review

**DOI:** 10.3390/jcm15176671

**Published:** 2026-08-28

**Authors:** Karol Makiel, Agnieszka Suder

**Affiliations:** Department of Anatomy, Faculty of Physical Rehabilitation, University of Physical Culture, 31-571 Cracow, Poland

**Keywords:** obesity, metabolic syndrome, resistance training, active recovery, combined exercise, gut microbiota, DNA methylation, adipokines, multi-omics

## Abstract

**Background/Objectives:** Resistance training with aerobic active recovery (RT-AR) is a proposed session architecture in which controlled low-impact aerobic activity replaces part of passive inter-set recovery. This hypothesis-driven narrative review evaluates its rationale in adults with obesity and metabolic syndrome (MetS) and proposes a multi-omics research framework. **Methods:** Structured searches of PubMed/MEDLINE and Scopus were combined with a narrative synthesis of systematic reviews, meta-analyses, controlled intervention studies, and mechanistic studies. Dedicated high-sensitivity searches were performed for studies directly evaluating aerobic active recovery during resistance-training inter-set recovery. **Results:** No randomized chronic intervention studies directly evaluating RT-AR in adults with obesity or MetS were identified; therefore, the rationale presented here is based on indirect evidence from conventional exercise and mechanistic studies. Evidence from overweight, abdominal-obesity, insulin-resistance, or type 2 diabetes cohorts was treated as partially applicable or indirect when the target phenotype differed. Aerobic, resistance, and combined training support improvements in body composition, cardiometabolic outcomes, physical function, and selected adipokines, whereas microbiota and DNA-methylation findings remain heterogeneous and are not RT-AR-specific. **Conclusions:** RT-AR should be regarded as a testable training architecture rather than an established therapeutic modality. Future trials should determine whether its effects exceed those attributable to total exercise dose, whether the resistance-training stimulus is preserved, and how diet, medication use, sex, and other major modifiers influence clinical and multi-omics responses.

## 1. Introduction

Metabolic syndrome (MetS) is defined by the clustering of abdominal obesity, impaired glucose regulation/insulin resistance, dyslipidemia, and elevated blood pressure, with increased cardiometabolic risk [1]. Obesity is a chronic metabolic disease in which excess adiposity can coexist with adipose-tissue dysfunction, ectopic lipid accumulation, altered adipokine secretion, and chronic low-grade inflammation [2,3,4,5]. Gut dysbiosis and epigenetic regulation, including DNA methylation, have also been implicated in the metabolic heterogeneity of obesity and related disorders [6,7,8]. Physical inactivity and excessive sedentary time further contribute to cardiometabolic risk, making structured physical activity a central component of prevention and treatment [9,10,11].

Exercise is a core component of obesity and MetS management. Aerobic training has traditionally been emphasized because of its effects on energy expenditure, cardiorespiratory fitness, and adiposity [12], whereas current guidance also recommends regular muscle-strengthening activity [9,10]. Resistance training is particularly relevant for preserving or increasing muscle mass and strength, while comparative evidence suggests that combined aerobic–resistance approaches can provide broader benefits across body composition, functional capacity, and selected cardiometabolic outcomes than either modality alone in some populations and study designs [13,14].

For clarity, the primary target population of this review is adults with obesity and/or MetS. These phenotypes overlap but are not interchangeable: obesity is defined primarily by excess adiposity, whereas MetS reflects a clustering of cardiometabolic abnormalities and may occur across different degrees of adiposity [1,2]. Throughout the review, evidence from adults with obesity or MetS is treated as directly applicable; evidence from overweight or abdominal-obesity cohorts without confirmed MetS is considered partially applicable; and evidence from insulin-resistant or type 2 diabetes cohorts without the target phenotype, as well as from healthy or athletic populations, is treated as indirect unless otherwise specified.

Despite established evidence supporting aerobic, resistance, and combined exercise in obesity and MetS [13,14,15,16,17,18,19], considerably less attention has been given to how these components are organized within individual training sessions. RT-AR represents a specific session architecture in which controlled low-impact aerobic activity is embedded within resistance-training inter-set recovery rather than performed as a separate exercise block. The conceptual distinction therefore lies not in combining resistance and aerobic exercise per se, but in the temporal placement of the aerobic component within inter-set recovery. This architecture warrants separate investigation because its safety, feasibility, and efficacy in adults with obesity and/or MetS have not yet been established.

For the purposes of this review, RT-AR is operationally defined as resistance training in which planned low-impact aerobic activity is performed within the inter-set recovery interval—after a working resistance set and before the subsequent working set—while resistance exercise remains the primary training stimulus. The aerobic component is performed at a controlled low-to-moderate intensity and is individualized so that it does not materially compromise subsequent resistance-training performance; detailed candidate parameters are presented in the proposed protocol section. RT-AR is distinguished from merely shortening passive rest, conventional sequential aerobic–resistance training, circuit training, and active-rest approaches in which another resistance, mobility, or stretching task is performed between sets.

Accordingly, this article is a narrative, hypothesis-generating review with a conceptual and translational purpose rather than a systematic review of RT-AR efficacy. It critically synthesizes evidence from aerobic, resistance, and combined exercise together with mechanistic literature to establish the rationale for RT-AR, define the proposed training architecture, and identify priorities for experimental validation. Dedicated searches were additionally performed to determine whether chronic intervention studies meeting the operational RT-AR definition could be identified.

## 2. Methods for Literature Search

This article is a narrative, hypothesis-generating review with a conceptual and translational focus. A structured literature search was used to support the narrative synthesis, identify the current evidence base, and define research gaps relevant to the proposed RT-AR concept. The review was not designed or conducted as a systematic review of RT-AR efficacy. The search strategy was developed to identify current systematic reviews and meta-analyses examining the effects of different exercise modalities used in obesity and metabolic syndrome and to perform dedicated high-sensitivity searches for studies meeting the operational definition of RT-AR. Particular emphasis was placed on publications concerning aerobic training, resistance training, interval training, combined training, and protocols in which the structure of the training session was manipulated, including the nature of recovery periods between exercise sets.

Electronic searches were conducted in PubMed/MEDLINE and Scopus. The broad evidence-mapping stage was designed to identify recent systematic reviews and meta-analyses of aerobic, resistance, interval, concurrent, and combined exercise in adults with obesity or metabolic syndrome. The original PubMed/MEDLINE search, limited to the preceding 10 years and to systematic reviews/meta-analyses, yielded 696 records. A database-adapted Scopus search using title terms for exercise and obesity/MetS, systematic-review/meta-analysis terms in TITLE-ABS-KEY, and PUBYEAR > 2015 yielded 586 records. Because this article is a narrative review, these broad searches were used to map the evidence base and prioritize higher-level evidence rather than to create a pooled PRISMA-style screening dataset. Exact database-specific search strings, limits, dates, and yields are provided in Table S1 (Supplementary Materials).

A separate high-sensitivity search was performed specifically to test the central research-gap statement concerning RT-AR. To avoid relying on the title-restricted broad search, active-recovery terminology was searched in Title/Abstract fields in PubMed/MEDLINE and in TITLE-ABS-KEY in Scopus, together with resistance/strength-training terms. The unrestricted-population searches yielded 270 records in PubMed/MEDLINE and 158 records in Scopus and were used to identify acute proof-of-concept studies and terminology related to inter-set active recovery. A prespecified population-targeted search adding overweight, obesity, abdominal obesity, and metabolic-syndrome terms yielded 14 PubMed/MEDLINE and 17 Scopus records. Eleven duplicates were removed by DOI and normalized-title matching, leaving 20 unique records for screening.

For the dedicated population-targeted RT-AR search, an eligible direct study was defined a priori as a primary human study in which a resistance-exercise set was followed by planned aerobic activity during the inter-set recovery period (e.g., treadmill walking/running, stationary cycling, or another low-impact aerobic modality) before the next resistance set. For the main clinical research-gap claim, the population had to comprise adults with overweight, obesity, abdominal obesity, or metabolic syndrome and the intervention had to evaluate a repeated/chronic training program. Studies were excluded when ‘active recovery’ consisted only of low-load resistance repetitions or stretching; when the aerobic component was performed as a separate sequential block; when active recovery occurred only between aerobic/HIIT intervals; when circuit training or HIFT did not explicitly embed aerobic recovery between resistance sets; or when the study was pediatric, non-human, a review, editorial, or protocol without relevant intervention data. Of the 20 unique records, 17 were excluded after title/abstract screening and 3 potentially relevant reports underwent full-text assessment. All 3 were excluded: two used low-load resistance repetitions as active rest with aerobic exercise performed in a separate sequential block, and one used stretching between resistance stations while aerobic active recovery occurred only within a subsequent aerobic block. On this basis, we did not identify any randomized chronic intervention study meeting the operational RT-AR definition in adults with overweight, obesity, or MetS.

Screening of the dedicated RT-AR search was performed by K.M. and subsequently verified by A.S.; uncertainties regarding eligibility were resolved by discussion and consensus. Duplicate removal was based on DOI and title matching, and full texts were checked when the intervention structure could not be determined reliably from the title and abstract. For the broader narrative synthesis, formal dual-independent systematic-review screening was not applied. Instead, studies were selected according to relevance to the predefined domains and a hierarchy favoring systematic reviews/meta-analyses, randomized or controlled intervention studies, and then mechanistic primary studies. Adult obesity/MetS evidence and studies most directly comparable with combined training or RT-AR were prioritized. Pediatric studies were excluded from the clinical synthesis; preclinical or cellular studies were used only when required to support a specific mechanistic point and were not treated as evidence of clinical RT-AR efficacy. Reference lists of key reviews and directly relevant primary studies were also checked manually; no formal citation-network analysis was used.

For interpretation of the narrative evidence, population applicability was also considered explicitly: studies conducted in adults with obesity or MetS were treated as direct evidence for the target phenotype; overweight or abdominal-obesity cohorts without confirmed MetS were considered partially applicable; and studies centered on type 2 diabetes, insulin resistance, healthy adults, or trained populations were treated as indirect unless participants also met the target obesity/MetS criteria.

Domain-specific PubMed/MEDLINE searches were performed to make the microbiota and epigenetic sections reproducible. For the gut microbiota domain, the broad exercise–microbiota–obesity/MetS search yielded 541 records; restriction to systematic reviews/meta-analyses yielded 18 records; a broader intervention/mechanistic layer yielded 197 records; and restriction to randomized/controlled/clinical trials yielded 43 records. For DNA methylation/epigenome, the corresponding broad search yielded 250 records, with 6 systematic reviews/meta-analyses and 15 randomized/controlled/clinical trials. A focused *ADIPOQ*/*LEP*–methylation–exercise search yielded 33 records, of which 23 remained after addition of obesity/MetS terms. These layered searches were used for evidence mapping and targeted narrative selection rather than as separate systematic-review datasets. In the Supplementary Materials, Table S1 provides the exact strings and yields, Table S2 provides the eligibility framework, and Table S3 summarizes the dedicated RT-AR screening flow.

The initial PubMed/MEDLINE evidence-mapping search was last updated on 11 August 2026. The additional Scopus search and all dedicated reproducibility searches performed in response to peer review were conducted on 20 August 2026. Search results were interpreted in accordance with the narrative, hypothesis-generating purpose of the review: the structured searches were used to establish the evidence base, test the absence of direct chronic RT-AR evidence in the target population, and support transparent selection of mechanistic literature, rather than to estimate pooled RT-AR efficacy.

### Research Gap and Multi-Omics Rationale for the RT-AR Concept

The structured literature search showed that current systematic reviews and meta-analyses of exercise interventions in individuals with obesity or metabolic syndrome focus primarily on aerobic training, resistance training, high-intensity interval training, combined training, and diet–exercise interventions. Most available protocols use a conventional combined-training model in which aerobic and resistance components are performed as separate parts of the same session or as separate sessions [15,16,17,18,19].

In the cited network meta-analysis, aerobic training ranked highest for reductions in body weight, BMI, and waist circumference, whereas combined training ranked favorably for fat mass and, similarly to resistance training, for fat-free mass [15]. These rankings are specific to the populations, interventions, comparators, durations, and outcomes included in that analysis and should not be interpreted as universally applicable superiority.

Interventions combining high-intensity aerobic exercise with high-load resistance training may produce favorable effects on abdominal adipose tissue, fat-free mass, and cardiorespiratory fitness [16].

A meta-analysis including 81 randomized trials indicated that same-session combined resistance and aerobic exercise ranked among the more favorable strategies for improving overall cardiometabolic health in adults with overweight or obesity within the comparisons included in that analysis [18].

Regular exercise may also reduce visceral adipose tissue. In the cited meta-analysis, larger average reductions were observed with high-intensity aerobic exercise and HIIT, whereas resistance training alone showed smaller effects for this outcome [19].

A limited number of protocols also incorporate activity during inter-set recovery; however, these most often involve light resistance work, circuit-based strategies, or acute exercise responses rather than a controlled, low-impact aerobic component systematically embedded between resistance-training sets [20,21,22,23]. Acute aerobic active recovery has been examined directly, including treadmill exercise between bench-press sets, but not as a chronic intervention in adults with obesity or metabolic syndrome [24]. These active inter-set recovery studies, together with passive-recovery resistance training, conventional concurrent/combined training, and circuit training, are the primary comparators for RT-AR and are discussed further in Section 6.3. CrossFit/HIFT is mentioned only to clarify terminology: it typically uses variable, higher-intensity multimodal or circuit-based structures rather than standardized low-impact aerobic recovery inserted specifically between resistance sets [25,26]. Musculoskeletal considerations associated with obesity are addressed separately as indirect safety evidence [27].

In the dedicated PubMed/MEDLINE and Scopus searches, we did not identify any randomized chronic intervention studies meeting the operational definition of RT-AR used in this review in adults with overweight, obesity, or metabolic syndrome. The control search combining exercise interventions with omics and multi-omics terms likewise did not identify an evidence synthesis directly integrating this specific training architecture with the gut microbiota–DNA methylation–adipokine axis.

The research gap therefore has two dimensions: the absence of chronic clinical validation of the RT-AR architecture in adults with overweight, obesity, or metabolic syndrome, and the absence of its evaluation from a multi-omics perspective. Acute inter-set active-recovery experiments in other populations provide proof-of-concept for the session architecture but do not establish its long-term clinical efficacy. Accordingly, the novelty proposed in this review does not concern the invention of aerobic inter-set recovery itself; rather, it concerns the systematic incorporation of this architecture into a progressive chronic training model for adults with obesity and/or MetS and its comparator-controlled clinical and multi-omics evaluation. This review is conceptual and translational in nature and integrates evidence on aerobic, resistance, and combined training with literature concerning the gut microbiota, DNA methylation, adipokines, and myokines. Its objective is to formulate a biological rationale and a testable protocol framework for future intervention studies rather than to demonstrate superiority of RT-AR over existing exercise modalities.

Accordingly, the evidence base informing RT-AR should be interpreted at three distinct levels: (1) direct chronic evidence for RT-AR in the target population; (2) indirect clinical evidence derived from established aerobic, resistance, and combined exercise interventions; and (3) mechanistic evidence used to formulate testable molecular hypotheses. Table 1 therefore organizes the literature according to its directness in relation to the proposed RT-AR model rather than assigning qualitative strength-of-evidence ratings. This hierarchy does not represent a formal assessment of methodological quality or certainty of evidence.

## 3. Clinical and Adipokine Outcomes Relevant to the RT-AR Concept

Regular exercise in adults with overweight, obesity, or metabolic syndrome favorably affects major components of cardiometabolic risk, although the magnitude of benefit depends on the exercise modality used. Aerobic training has particularly well-documented effects on reductions in body weight, BMI, waist circumference, and visceral adipose tissue and, in patients with metabolic syndrome, is also associated with improved cardiorespiratory fitness, lower blood pressure, reduced fasting glucose and triglyceride concentrations, and increased HDL-C [17,28,29]. From this perspective, aerobic training remains a fundamental intervention when the principal goals are to reduce central obesity, improve fitness, and lower blood pressure and cardiovascular risk.

Resistance training is particularly important for preserving fat-free mass during weight loss, improving muscle strength, and maintaining skeletal-muscle metabolic function [28]. Bodybuilding populations provide an illustrative example of the high-muscle/low-fat phenotype achievable with sustained resistance training [30], but they are not direct evidence for obesity or MetS treatment. Resistance training may also improve fat mass, LDL-C, and systolic blood pressure, although its average metabolic effects are narrower than those reported for combined training in the cited analyses [17,31].

Across the cited meta-analyses, combined training has shown broad benefits across several components of metabolic syndrome, including glycemic control, insulin resistance, triglyceride concentrations, waist circumference, blood pressure, and body composition [17,31]. These findings support combined training as a well-supported option when multiple cardiometabolic and functional outcomes are targeted simultaneously, without implying universal superiority across all populations or protocols.

Adipose tissue and skeletal muscle form an integrated endocrine communication network involving adipokines, myokines, and extracellular vesicles that participate in the regulation of inflammatory responses, glucose homeostasis, and lipid metabolism [32,33]. Meta-analyses indicate that regular exercise may modulate CRP, TNF-α, leptin, and adiponectin, whereas the response of IL-6 and other markers depends on the population and intervention characteristics [34,35]. Additional evidence comes from a series of related intervention studies in men with metabolic syndrome or abdominal obesity in which irisin, IL-6, adiponectin, IL-8, leptin, omentin, and asprosin were examined in relation to body composition and carbohydrate and lipid metabolism [36,37,38,39,40,41,42]. Because these publications analyze different outcomes derived from related research protocols, they should be regarded as complementary mechanistic analyses rather than independent replications of the same effect. Because several studies discussed in this section originated from the authors’ research group and related intervention protocols [36,37,38,39,40,41,42], these findings were interpreted alongside broader systematic reviews, meta-analyses, and independent intervention evidence [34,35] to minimize the risk of selective interpretation. They were not treated as independent replications or as the sole evidence supporting the RT-AR rationale.

The most consistent signal concerned leptin and asprosin. In men with metabolic syndrome, aerobic–resistance training produced a more pronounced reduction in leptin than aerobic training, and leptin concentrations remained strongly associated with total and android fat mass [38]. In a protocol combining aerobic–resistance training with a high-protein diet based on low-glycemic-index foods, the reduction in leptin was greater than after exercise alone, while asprosin concentrations also decreased in both intervention groups [41]. These findings suggest that the response of these adipokines is closely linked to changes in adipose tissue, lipid metabolism, and the quality of the dietary intervention, thereby limiting attribution of the effect to exercise modality alone.

The responses of adiponectin and omentin were less consistent. A comparison of aerobic with aerobic–resistance training showed no significant change in adiponectin despite improvements in selected indices of insulin resistance, waist circumference, and fat-free mass [37]. By contrast, adding a nutritional intervention to aerobic–resistance training was associated with an increase in adiponectin together with reductions in IL-6, hs-CRP, and selected indices of insulin resistance and atherogenicity [42]. Omentin did not show a consistent response to short-term interventions despite observed associations with some lipid parameters [38,40]. Taken together, these findings support viewing the adipokine profile as part of a broader metabolic-response phenotype rather than as an isolated mechanism of RT-AR action [37,38,39,40,41,42]. From a multi-omics perspective, adipokines should therefore be analyzed in parallel with body composition, glycemia, inflammatory markers, and the gut microbiota and its metabolites [43,44,45]. Because several adipokine intervention studies summarized here enrolled men, these findings should not be assumed to generalize identically to women; sex should be considered a potential effect modifier in future RT-AR analyses.

## 4. Gut Microbiota as a Potential Target of RT-AR: Indirect Evidence

Within the targeted literature searches, we did not identify any chronic intervention study meeting the operational RT-AR definition that evaluated gut microbiota outcomes in individuals with overweight, obesity, or metabolic syndrome. RT-AR should therefore not be presented as a method that induces a specific remodeling of the microbiota, but rather as a potential extension of combined training that may indirectly influence the intestinal environment through changes in energy expenditure, body composition, insulin sensitivity, inflammation, and skeletal-muscle metabolic function. The microbiota forms part of a bidirectional gut–adipose tissue–muscle axis in which the host metabolic state influences the microbial environment, while the composition and metabolic activity of the microbiota may modulate intestinal barrier function, immune responses, adipokine secretion, and glucose and lipid metabolism [43,44]. For clarity, the section proceeds from evidence derived from exercise studies, through findings specifically relevant to obesity/MetS, to explicitly hypothesized mechanisms and priorities for future RT-AR testing.

### 4.1. Evidence from Exercise Studies and Obesity

Current systematic reviews and meta-analyses suggest that regular physical exercise can alter gut microbiota structure in individuals with obesity, although the magnitude and direction of these changes are heterogeneous. In a meta-analysis of randomized trials by Kim et al., exercise significantly affected beta diversity, indicating a shift in overall bacterial community structure; however, the number of studies allowing quantitative synthesis of individual indices was limited [45]. In contrast, the review by Cancino Ramírez et al. did not confirm a consistent effect of exercise on alpha diversity but reported obesity-status-dependent changes in beta diversity and positive associations between moderate- or vigorous-intensity activity and the presence of short-chain fatty acid-producing bacteria [46]. These discrepancies indicate that the microbiota response to exercise is more complex than a simple increase in diversity and may primarily involve functional and metabolic changes and altered relationships among individual taxa.

Exercise modality may also be relevant. Most available data concern aerobic interventions lasting from several weeks to several months. A broad review of intervention studies suggested that moderate- or vigorous-intensity exercise totaling approximately 150–270 min per week for at least eight weeks may increase the likelihood of detecting microbiota changes [47]. This does not, however, establish a clear dose–response relationship, because outcomes are also influenced by diet, baseline fitness, age, body composition, medication use, sequencing methods, and bioinformatic approaches. A meta-analysis in adults showed only a small increase in the Shannon index, supporting the view that the average effect of exercise on alpha diversity may be modest [48].

In the study by Allen et al. [49], six weeks of endurance training altered the composition and metabolic potential of the gut microbiota, but the response depended on adiposity. Increases in fecal short-chain fatty acid concentrations were observed mainly in participants with normal body weight, whereas changes were less pronounced in participants with obesity. These findings suggest that the obesity phenotype may attenuate or modify the microbiota response to exercise; therefore, observations from lean or trained individuals should not be directly extrapolated to patients with obesity and metabolic syndrome. After the intervention ended and participants returned to a sedentary lifestyle, the microbiota profile partially shifted toward baseline, further suggesting that maintenance of these changes may require continued physical activity.

A comparison of sprint interval training with continuous moderate-intensity aerobic training in individuals with insulin resistance showed that both modalities reduced TNF-α and LPS-binding protein concentrations and modified the microbiota profile. However, only continuous training reduced fatty acid uptake in the jejunum, whereas the increase in cardiorespiratory fitness was more pronounced after interval training [50]. These findings suggest that different forms of exercise may affect distinct components of the gut–metabolic axis and that higher intensity does not necessarily produce a stronger microbiota response or a more favorable change in intestinal metabolism.

Data on resistance training are substantially more limited. In a six-week study of individuals with excess body weight, resistance training increased the relative abundance of the genus Roseburia, which includes butyrate-producing bacteria, but did not significantly alter overall microbiota diversity. Only modest shifts in predicted bacterial metabolic pathways were observed [51]. This may indicate that the microbiota response to resistance training concerns selected taxa or metabolic functions rather than broad community remodeling. The study does not, however, establish whether these changes resulted directly from the resistance stimulus, increased muscle activity, changes in dietary behavior, or improvement in host metabolism.

Studies of combined training represent the most relevant indirect comparator for the RT-AR concept. The most recent meta-analysis including individuals with obesity or type 2 diabetes reported increases in the Shannon and Chao1 indices, with no significant changes in the Simpson index or the number of observed taxonomic units. In subgroup analyses, combined training produced a more pronounced improvement in the Shannon index than aerobic training alone in both individuals with obesity and patients with type 2 diabetes. The qualitative synthesis also repeatedly identified increases in butyrate-producing bacteria, including Roseburia and Faecalibacterium prausnitzii, as well as Akkermansia muciniphila, whereas changes in the Bacillota/Bacteroidota ratio and in Prevotella and Bacteroides were inconsistent [52]. These data suggest that combining aerobic and resistance components may affect selected microbiota features, but they do not establish a uniform response profile or the efficacy of aerobic active recovery between sets. The subgroup analyses included a limited number of studies and should therefore be interpreted cautiously.

From an RT-AR perspective, it is important to distinguish three possible roles of the microbiota. Microbiota changes may act as a mediator of metabolic improvement if they participate in mechanisms leading to changes in glycemia, inflammation, or the adipokine profile. The microbiota may also act as a modifier when its baseline composition and activity influence the magnitude of the training response, or merely as a marker that changes in parallel with metabolic improvement without a demonstrated causal role. This distinction helps organize interpretation of the available evidence and emphasizes that a change in bacterial abundance alone is insufficient to establish a mechanism of exercise action.

Favorable changes in body composition, insulin sensitivity, and inflammation, discussed in the preceding section, may indirectly modify the metabolic environment in which the gut microbiota operates [29,34,35,53]. Physical activity also affects gastrointestinal physiology, including motility, transit time, splanchnic perfusion, intestinal barrier permeability, and nutrient absorption [54,55]. As exercise intensity increases, blood flow is redistributed toward working muscles, which may lead to transient splanchnic hypoperfusion, enterocyte injury, and increased small-intestinal permeability [55]. Prolonged or intense exercise performed under hyperthermic conditions may aggravate intestinal barrier disruption [56], whereas hypohydration may moderately increase epithelial injury and gastrointestinal dysfunction without consistently exacerbating endotoxemia [57].

Most of these observations, however, come from healthy or trained individuals. In patients with obesity and metabolic syndrome, in whom intestinal barrier dysfunction, chronic inflammation, and metabolic endotoxemia may already be present, the response to a similar exercise load may differ. This supports cautious progression of RT-AR intensity and direct assessment of exercise tolerance and markers of intestinal function in this population.

### 4.2. Hypothesized Mechanisms Relevant to RT-AR

The pathways discussed below—including SCFA–FFAR2/3, bile acid–FXR/TGR5, tryptophan–AhR, and LPS–TLR4 signaling—should be interpreted as hypothesis-generating mechanisms derived from general microbiota–host biology and exercise studies using other modalities. None has been demonstrated as an RT-AR-specific mechanism. Accordingly, these pathways should be treated as exploratory hypotheses to be tested rather than as assumed mediators of RT-AR.

The mechanistic relevance of the microbiota to the exercise response derives primarily from its functional activity and the metabolites it produces rather than simply from the presence of particular taxa. If RT-AR were to contribute to remodeling of the gut–metabolic axis, this effect could be expressed through changes in metabolite production, intestinal barrier integrity, and communication with adipose tissue and skeletal muscle, even in the absence of substantial changes in overall microbiota diversity.

Butyrate, propionate, and acetate, which are short-chain fatty acids (SCFAs), may influence intestinal epithelial integrity, appetite regulation, secretion of gut hormones, and glucose and lipid metabolism. Their actions are partly mediated through activation of G protein-coupled receptors 41 and 43 (GPR41 and GPR43), also known as free fatty acid receptors 3 and 2 (FFAR3 and FFAR2), respectively. Activation of these receptors may affect, among other processes, the secretion of gut hormones involved in satiety and glucose homeostasis, immune responses, and intestinal barrier function [58]. In exercise studies, increases in SCFA production were more evident in individuals without obesity, whereas the response was weaker in participants with obesity. This suggests that the baseline metabolic phenotype may modify the functional response of the microbiota to exercise and that effects observed in lean individuals should not be directly extrapolated to patients with obesity and MetS [49,58].

The gut microbiota also participates in bile acid transformation, thereby modifying their composition and signaling activity. Changes in the bile acid pool may influence signaling through the farnesoid X receptor (FXR) and G protein-coupled bile acid receptor 1 (TGR5). These receptors participate in the regulation of bile acid synthesis, glucose and lipid metabolism, gut hormone secretion, and systemic energy homeostasis [58]. Another mechanism involves bacterial metabolism of tryptophan. The resulting indole derivatives can activate the aryl hydrocarbon receptor (AhR), which participates in regulation of immune responses and maintenance of intestinal epithelial integrity. Activation of this pathway may support mucosal renewal and limit excessive inflammatory responses within the intestine [58].

The bile acid and tryptophan-derivative pathways illustrate how a functional microbiota response can influence host metabolism without requiring extensive changes in taxonomic composition. In studies of RT-AR, it would therefore be appropriate to assess not only which bacteria increase or decrease in abundance, but also whether the pool of metabolites acting on FXR, TGR5, and AhR changes.

A different, unfavorable mechanism is associated with increased translocation of bacterial lipopolysaccharide (LPS) across the intestinal barrier. LPS, a component of the outer membrane of Gram-negative bacteria, can activate Toll-like receptor 4 (TLR4)-dependent pathways. Activation of this receptor enhances pro-inflammatory signaling and cytokine secretion, which may impair insulin signaling, promote insulin resistance, and worsen adipose tissue function [58]. Bacterial metabolites may also affect the expression, secretion, and biological actions of leptin, adiponectin, and other adipokines. These effects may be direct, through actions of metabolites on adipocytes, or indirect, through changes in intestinal barrier integrity, LPS translocation, inflammation, and insulin sensitivity [43,58].

These mechanisms create a functional link among the gut, adipose tissue, skeletal muscle, and the immune system. Within such a model, the microbiota could mediate part of the adipokine and inflammatory changes induced by exercise, but it is equally plausible that improvements in body composition and insulin sensitivity secondarily alter the intestinal environment. Available studies do not yet allow the direction of this relationship to be established conclusively.

It should therefore not be assumed that every improvement in metabolic parameters is mediated by changes in the gut microbiota. In a randomized study, a three-week intervention combining energy restriction with vigorous treadmill walking significantly reduced body weight, fat mass, and insulin, leptin, and cholesterol concentrations and improved insulin sensitivity, while producing no measurable changes in microbiota diversity, taxonomic composition, or functional potential [59]. These findings indicate that early metabolic improvement can occur independently of detectable microbiome remodeling. This does not exclude a later microbiota response or changes occurring with a longer intervention, a different exercise dose, or concurrent qualitative modification of the diet.

### 4.3. What Remains Unknown and Priorities for Future RT-AR Trials

For RT-AR, the most cautious hypothesis is that replacing passive recovery with controlled aerobic exercise may increase the total volume of aerobic activity, reduce time spent inactive, and augment the metabolic benefits of resistance training without extending the session by adding a separate aerobic block. Any microbiota changes could be part of the mechanism of metabolic improvement or could occur secondarily to that improvement. The baseline microbiota profile may also modify the magnitude of the training response even if the microbiota itself does not undergo substantial remodeling. Potential effects on the microbiota would probably be linked to changes in body composition, glycemic control, insulin sensitivity, adipokine profile, and inflammation rather than to a specific effect of the active-rest structure itself. Current evidence does not allow identification of an RT-AR-specific microbiota profile.

From a multi-omics perspective, evaluation of RT-AR should not rely solely on 16S rRNA analysis; preferentially, it should incorporate shotgun metagenomics combined with fecal and plasma metabolomics. Particular attention should be given to SCFAs, secondary bile acids, tryptophan derivatives, and markers of endotoxemia, analyzed in parallel with body composition, insulin sensitivity, lipid profile, inflammatory markers, and the adipokine profile.

Such an approach could help distinguish whether microbiota changes precede metabolic improvement and may therefore act as mediators, or whether they simply occur in parallel as markers of the training response. Analysis of the baseline microbiome could also determine whether a particular functional profile enhances or limits the response to RT-AR, thereby acting as an effect modifier. Resolving these relationships will require repeated measurements over time and integration of microbiota data with metabolites, adipokines, inflammatory markers, and clinical parameters. Before any of these pathways is interpreted as a mediator, future trials must first establish that RT-AR modifies the relevant microbiome or metabolite endpoint at all.

Microbial metabolites may also influence the availability of substrates and cofactors involved in chromatin regulation, providing a transition to the next level of multi-omics analysis—DNA methylation.

## 5. DNA Methylation and the Epigenome: Evidence Relevant to the RT-AR Hypothesis

Within the targeted literature searches, we did not identify any chronic intervention study meeting the operational RT-AR definition that evaluated DNA methylation in individuals with overweight, obesity, or metabolic syndrome. RT-AR should therefore not be presented as a method that induces a specific epigenetic profile. Its potential biological effects can only be inferred indirectly from studies of acute exercise responses, aerobic, resistance, and combined training, and changes observed in skeletal muscle, adipose tissue, and blood cells.

Accordingly, all epigenetic pathways and loci discussed below should be interpreted as exploratory hypotheses derived from other exercise modalities and metabolic studies; no RT-AR-specific DNA-methylation profile or validated epigenetic target has been established.

DNA methylation in skeletal muscle can respond rapidly to an exercise stimulus. Following a single exercise session, intensity-dependent reductions in methylation of promoter regions of genes involved in energy metabolism, including *PPARGC1A*, *PDK4*, and *PPARD*, were accompanied by increased gene expression [60]. Methylation changes may therefore participate in the early transcriptional response to muscle contraction, but findings from an acute exercise bout do not establish whether these changes persist across repeated sessions or contribute to long-term metabolic improvement.

Repeated exercise training may lead to broader remodeling of the skeletal-muscle epigenome. After three months of endurance training, coordinated changes in DNA methylation and gene expression were observed in genes involved, among other processes, in energy metabolism, mitochondrial function, and inflammatory responses [61]. A six-month exercise intervention also modified methylation of genes involved in insulin and calcium signaling, substrate metabolism, and the respiratory chain in skeletal muscle from men with or without a family history of type 2 diabetes. For *ADIPOR1*, *BDKRB2*, and *TRIB1*, methylation changes were accompanied by changes in gene expression [62]. These findings confirm substantial plasticity of the skeletal-muscle epigenome; however, the direction of methylation changes depends on the type and duration of the intervention, the genes examined, and the timing of biological sample collection.

Resistance-training studies are also relevant to the RT-AR concept. In a study involving hypertrophy-inducing training, detraining, and subsequent retraining, some methylation changes in skeletal muscle persisted despite muscle mass returning toward baseline. Retraining was accompanied by a larger number of hypomethylated CpG sites and increased expression of selected genes associated with muscle adaptation. The authors interpreted these observations as evidence of a potential epigenetic memory of previous resistance training [63]. Because the study included a small group of healthy men, lacked a parallel control group, and was exploratory in nature, its findings cannot be directly extrapolated to individuals with obesity, metabolic syndrome, or RT-AR. Nevertheless, it suggests that repeated resistance stimuli may leave relatively persistent signatures in the skeletal-muscle methylome.

Epigenetic changes may also occur in adipose tissue. After a six-month exercise intervention, widespread DNA methylation changes were identified in subcutaneous adipose tissue, involving genes related to obesity, type 2 diabetes, lipid metabolism, and adipocyte function. At some loci, methylation changes were accompanied by altered gene expression, suggesting that epigenetic mechanisms may participate in exercise-induced remodeling of adipose tissue function [64]. It remains unclear, however, to what extent these changes were caused directly by the exercise stimulus versus secondary improvements in body composition and metabolism.

One of the most relevant indirect comparators for the RT-AR concept was a 14-week combined-training program conducted in 41 older women with normal weight, overweight, or obesity. No change in global blood DNA methylation was observed, but 1043 differentially methylated CpG sites assigned to 744 genes were identified. The changes involved genes related to cellular metabolism, oxidative phosphorylation, and mitochondrial function; in women with overweight, they also included reduced methylation of eight genes associated with lipogenesis. Improvements in blood pressure, lipid profile, and physical fitness were not accompanied by a change in epigenetic age [65]. This study supports the possibility that combined training can influence the methylome, but it cannot separate the effects of resistance and aerobic components or determine the importance of how inter-set recovery is organized.

Findings across studies remain heterogeneous. A systematic review including 12 randomized trials and 827 previously inactive adults found that most interventions lasting from 6 weeks to 12 months produced changes in global methylation or methylation at selected loci. The response, however, depended on age, sex, health status, exercise type and duration, tissue analyzed, and laboratory method [66]. No universal methylation profile has therefore been identified for aerobic, resistance, or combined training.

In obesity and metabolic syndrome, interpretation of DNA methylation changes should account for baseline adipose tissue dysfunction. Methylation and gene-expression patterns in this tissue are associated with age, BMI, and HbA1c concentrations, and some differences involve genes participating in adipogenesis, lipid metabolism, insulin signaling, and inflammatory responses [67]. This suggests that the baseline epigenetic profile of individuals with obesity may influence their response to training. The relationship may be bidirectional: metabolic dysfunction can modify DNA methylation, while persistent epigenetic changes may contribute to maintenance of an unfavorable metabolic phenotype.

The *ADIPOQ* and *LEP* genes may be particularly relevant to epigenetic regulation of adipose tissue function. In an experimental study, obesity-associated activation of DNMT1 and hypermethylation of a specific region of the *ADIPOQ* promoter reduced its expression and contributed to insulin resistance. Relationships between *ADIPOQ* promoter methylation and expression were also evaluated in human adipocytes [68]. In addition, a 36 h fast altered *ADIPOQ* and *LEP* methylation in subcutaneous adipose tissue of adult men, with responses depending on birth weight [69]. These findings indicate that adipokine-related loci may respond to changes in energy availability, but they do not provide direct evidence regarding physical activity.

Intervention data show that the metabolic benefits of exercise do not necessarily result from changes in *ADIPOQ* and *LEP* expression. In men with obesity, three months of dynamic resistance training increased insulin sensitivity by 24% and reduced leptin concentrations by 21% without altering *ADIPOQ* or *LEP* mRNA expression in subcutaneous adipose tissue [70]. Similarly, 12 weeks of combined training comprising two aerobic and one resistance session per week improved fitness, muscle strength, and peripheral insulin sensitivity in men with obesity without altering expression of either gene [71]. Metabolic improvement can therefore occur without detectable changes in expression of these genes in subcutaneous adipose tissue.

The gene-expression responses of *ADIPOQ* and *LEP* are not uniform, however. In a randomized study of individuals with obesity, expression of *ADIPOQ* and adiponectin receptors in adipose tissue increased after exercise alone, a low-energy diet, and the combination of both interventions. Circulating adiponectin increased only in the groups undergoing energy restriction [72]. In older adults with overweight and abdominal obesity, increased physical activity was accompanied by increased *LEP* expression in adipose tissue despite a simultaneous reduction in circulating leptin [73]. mRNA expression therefore does not necessarily directly reflect adipokine secretion, which is also influenced by post-transcriptional regulation, translation, secretion, protein clearance, and adipocyte size and function.

Evidence on the direct effect of exercise on *ADIPOQ* and *LEP* methylation in adults with obesity or metabolic syndrome remains insufficient. In the focused search, we did not identify studies that simultaneously assessed methylation of these genes, their expression in adipose tissue, and changes in circulating adiponectin and leptin after isolated resistance training or aerobic–resistance training. *ADIPOQ* and *LEP* should therefore be treated as biologically plausible candidate loci for future RT-AR studies rather than as established epigenetic targets of this training model.

Epigenetic clocks represent a separate issue. They use methylation at selected CpG sites to estimate biological age or the rate of aging. Observational studies have reported associations among physical activity, BMI, metabolic syndrome parameters, and epigenetic age acceleration [74]. Newer algorithms, such as DNAm PhenoAge, were developed to better reflect disease risk and healthspan than clocks designed mainly to predict chronological age [75]. In RT-AR studies, such measures could serve as exploratory endpoints but should not be considered primary efficacy outcomes of a short-term intervention. The absence of a change in epigenetic age does not exclude changes at individual metabolic loci, as illustrated by the 14-week combined-training study [65].

For RT-AR, it can be hypothesized that replacing passive recovery with controlled aerobic exercise increases the continuity of the contractile–metabolic stimulus. This model may combine signals generated by mechanical tension with sustained ATP demand and oxidative processes, potentially influencing regulation of genes associated with mitochondrial function, glucose transport, lipid metabolism, and muscle adaptation. This remains a translational hypothesis. There is currently no basis for claiming that RT-AR induces stronger, more persistent, or more favorable DNA methylation changes than conventional resistance, aerobic, or sequential combined training.

Based on indirect evidence from exercise and metabolic studies, the loci listed below can be considered hypothesis-driven candidate targets for exploratory analysis; their methylation response to RT-AR remains entirely unknown.

Future RT-AR studies could reasonably incorporate whole-methylome analysis supplemented by assessment of selected gene-expression targets. Genes of particular interest may include *PPARGC1A*, *PDK4*, and *PPARD*, which are associated with energy metabolism; *IRS1* and *SLC2A4*, which participate in insulin signaling and glucose transport; and *ADIPOQ*, *LEP*, and *PPARG*, which are related to adipose tissue function. Selection of loci should be based on prespecified biological hypotheses rather than on the assumption that hypermethylation or hypomethylation is inherently beneficial or detrimental. Accordingly, these loci should be regarded as prespecified exploratory targets rather than validated RT-AR biomarkers or mechanistic targets.

Importantly, histone modifications and DNA methylation are distinct epigenetic mechanisms. Evidence that SCFAs influence histone deacetylase activity or histone acetylation should not be interpreted as evidence that they induce DNA methylation changes. Any proposed link between microbiota-derived metabolites and RT-AR-related DNA methylation would require direct measurement of both metabolite exposure and methylation at relevant CpG sites.

The greatest mechanistic value may come from integrating DNA methylation with gene expression, microbiota, bacterial metabolites, adipokines, inflammation, and the metabolic phenotype. Microbiota-derived metabolites, particularly butyrate and other SCFAs, can influence histone acetylation through inhibition of histone deacetylases [43,58]; this represents a distinct epigenetic mechanism from DNA methylation and should be treated as a parallel pathway rather than evidence of methylation change. Only studies that directly measure metabolite exposure together with DNA methylation at relevant CpG sites can test a microbiota–methylation relationship. Likewise, only direct comparison of RT-AR with conventional resistance training or dose-matched sequential combined training can determine whether active recovery provides a distinct biological signal or primarily changes total exercise dose.

## 6. Proposed RT-AR Protocol, Comparator Selection, and Safety Considerations

### 6.1. Translational Concept of Resistance Training with Aerobic Active Recovery

The proposed RT-AR model is neither conventional circuit training nor a sequential combination of separate resistance and aerobic components. Its defining feature is the replacement of passive inter-set recovery with short, controlled bouts of low-orthopedic-load aerobic activity while maintaining progressive resistance loading, correct exercise technique, and supervision. The illustrative research framework assumes three supervised sessions per week. Each session begins with approximately five minutes of low-intensity aerobic warm-up, followed by repeated sequences of a resistance-exercise set, aerobic active recovery, and the subsequent resistance set.

The protocol may be supervised individually or in small groups, provided that inter-set timing, exercise technique, physiological responses, and signs of intolerance can be monitored consistently.

Adults with obesity may spend more time sitting and may have lower habitual spontaneous activity than lean counterparts [76,77]. RT-AR can increase the amount of supervised movement performed within the training session by replacing part of passive inter-set recovery with controlled aerobic activity. This should not be interpreted as evidence that RT-AR increases non-exercise activity thermogenesis (NEAT) or reduces sedentary behavior outside structured exercise; compensatory changes in habitual activity remain possible and should be measured separately in future trials.

To reconcile reproducibility with the current absence of direct chronic RT-AR validation, the proposed framework is presented in two distinct layers. The first layer comprises general training principles supported by established resistance- and aerobic-exercise literature: progressive resistance loading, individualized submaximal intensity, low-impact aerobic activity during inter-set recovery, preservation of exercise technique, physiological and symptom monitoring, and gradual progression. The second layer comprises specific operational values proposed by the authors solely as candidate parameters for prospective experimental testing. These numerical settings should not be interpreted as evidence-based RT-AR prescriptions, optimal values, or an established clinical standard.

The musculoskeletal and exercise-selection considerations below should be regarded as pragmatic clinical considerations based on indirect evidence rather than as validated RT-AR-specific safety recommendations.

The rationale for exercise individualization is indirect and derives from literature showing that some individuals with obesity have a higher prevalence of low back pain, reduced trunk endurance, mobility limitations, abdominal-wall dysfunction, or reduced relative strength [78,79,80,81,82,83,84,85,86,87,88,89]. These findings support a cautious and progressive approach in which exercise complexity, external support, range of motion, and loading are adjusted to current functional capacity. They do not validate any single exercise sequence or BMI-specific prescription for RT-AR.

For reproducibility, Table 2 nevertheless specifies an illustrative candidate protocol that can be implemented and prospectively tested. It includes resistance-training intensity, volume, movement control, active-recovery modality, intensity and duration, progression rules, monitoring variables, and criteria for modifying or stopping exercise. The purpose of these details is experimental standardization, not clinical recommendation.

Accordingly, exercise selection should be based on functional assessment, symptoms, orthopedic tolerance, balance, lumbopelvic control, and technical competence rather than on a mandatory exercise list or fixed BMI threshold. The exercise variants shown in Table 3 are examples of possible adaptations only; other exercises serving the same movement pattern may be substituted when they improve safety, tolerance, or technique.

The potential value of RT-AR lies in combining several objectives within a single training session: reducing passive recovery time, increasing the aerobic component, maintaining the resistance stimulus needed to preserve muscle mass, and progressively developing trunk stabilization and gluteal function. This does not demonstrate superiority over sequential training or justify assuming greater efficacy in every patient with obesity. RT-AR should be treated as a coherent translational proposal whose safety, feasibility, and efficacy require testing in a randomized study incorporating clinical, metabolic, functional, and biomechanical outcomes.

The distinction between general principles and author-proposed test parameters is summarized in Table 2, while Table 3 presents general principles for individualizing exercise selection. Together, the tables are intended to make the candidate protocol reproducible for experimental validation without implying that its numerical settings have already been established as clinically optimal.

Safety monitoring should be based on symptoms, exercise technique, and physiological responses rather than on fixed BMI thresholds. Active recovery should be reduced in intensity or duration, replaced temporarily by passive recovery, or omitted when it causes disproportionate dyspnea, dizziness, loss of technique, inability to begin the subsequent resistance set at the planned effort, worsening musculoskeletal symptoms, or a clinically concerning heart-rate or blood-pressure response. The exercise session should be terminated if chest pain, presyncope or syncope, severe or disproportionate dyspnea, acute neurologic symptoms, or acute musculoskeletal pain occurs. Preparticipation assessment and supervision should be individualized according to symptoms, known disease, comorbidities, functional limitations, and planned exercise intensity; obesity or MetS alone should not be treated as an indication for routine exercise stress testing [90]. These criteria apply general clinical exercise-safety principles to a candidate research protocol and are not presented as RT-AR-specific validated thresholds.

This framework creates a coherent chain of proposed relationships but does not establish causality. In multi-omics projects, it is particularly easy to confuse covariation among biomarkers with a causal mechanism; therefore, interpretative limitations are an integral component of this review rather than merely a formal editorial addition.

### 6.2. The Critical Role of Exercise Dose and Comparator Selection

A central methodological issue in evaluating RT-AR is separating an effect of session architecture from an effect of greater total exercise dose. Replacing passive inter-set recovery with aerobic activity increases movement within the session and may increase aerobic minutes, cardiovascular exposure, and energy expenditure. Therefore, improvements in clinical or multi-omics outcomes could reflect a higher exercise dose rather than the temporal placement of aerobic activity itself.

Future trials should therefore compare (1) resistance training with passive inter-set recovery, (2) conventional sequential aerobic–resistance training, and (3) RT-AR. Resistance-training volume and progression should be standardized across groups, while RT-AR and sequential combined training should be matched as closely as possible for aerobic duration and relative intensity, total session duration, and, where feasible, energy expenditure.

These comparisons address different questions. RT-AR versus passive-recovery resistance training estimates the overall effect of adding aerobic activity during recovery, whereas RT-AR versus dose-matched sequential combined training tests whether the inter-set architecture has independent value. If an apparent advantage disappears after dose matching, the effect would be more plausibly attributed to exercise dose; persistence of a difference would provide stronger support for an architecture-specific effect.

An initial three-arm randomized trial should prespecify central adiposity/body composition and cardiometabolic outcomes, with cardiorespiratory fitness, strength, resistance-training performance, adherence, glycemic and lipid measures, blood pressure, inflammation, and adipokines as secondary outcomes. Microbiome, metabolomic, and DNA-methylation measures should initially remain exploratory. Studies should also quantify completed resistance volume, aerobic exposure, session duration, perceived exertion, heart-rate exposure, and energy expenditure, because matching prescribed exercise does not ensure that the delivered resistance stimulus is equivalent.

### 6.3. Potential Trade-Off Between Metabolic Continuity and Resistance-Training Performance

A second key issue is whether active recovery compromises the resistance stimulus. Acute findings are mixed rather than uniformly negative: low-intensity cycling did not impair force, power, or work in Mohamad et al. [91]; Scudese et al. found no reduction in total bench-press repetitions with treadmill active recovery at approximately 45% of VO_2_max [24]; and Corder et al. reported better subsequent repetition performance at 25% of the onset of blood lactate accumulation (OBLA) but not at 50% OBLA [92]. These findings suggest that effects depend on recovery intensity, exercise characteristics, and training status.

Inter-set recovery is itself an important determinant of resistance-training performance. Longer rest intervals may favor strength development in trained individuals [93] and may support hypertrophy in some settings by allowing greater volume-load to be maintained [94]. Thus, RT-AR could increase metabolic exposure while simultaneously weakening the resistance stimulus if it reduces repetitions, external load, movement velocity or power, technical quality, or total volume.

Because chronic RT-AR data are unavailable in adults with obesity or MetS, future trials should explicitly verify preservation of the resistance stimulus by recording repetitions, external load, volume-load, RIR/set and session RPE, adherence, and, where feasible, repetition velocity or power, together with longitudinal strength and lean-mass outcomes. Active-recovery intensity or duration should be reduced, or passive recovery temporarily restored, when planned workload or technique cannot be maintained; the aim is to identify a tolerable active-recovery dose that increases movement without materially compromising resistance training.

### 6.4. Personalized Nutrition and Artificial Intelligence/Machine Learning as Future Research Directions

Inter-individual responses to RT-AR may be influenced by diet, metabolic phenotype, gut microbiota, medication use, and other host factors. Personalized or precision-nutrition approaches could therefore be explored in future trials to test whether dietary characteristics modify clinical or molecular responses and to support prespecified stratification. These approaches are not established components of RT-AR, although large deeply phenotyped studies support the feasibility of integrating dietary, metabolic, and microbiome information in individualized prediction models [95,96].

Artificial intelligence and machine-learning methods may likewise help integrate high-dimensional clinical and multi-omics data to identify response patterns and explore predictors of benefit. Such applications remain exploratory and require adequate sample sizes, external validation, transparent modeling, control of overfitting and bias, interpretability, and appropriate data governance before they can inform individualized exercise or nutrition prescription [97].

## 7. Interpretative Limitations

The principal limitation is that, within the dedicated searches, we did not identify randomized chronic intervention studies meeting the operational RT-AR definition in adults with overweight, obesity, or metabolic syndrome. The current rationale for this concept is based on extrapolation from studies of aerobic, resistance, and combined training together with mechanistic evidence concerning the gut microbiota, adipokines, and DNA methylation. Consequently, the available evidence does not support conclusions that RT-AR is superior to conventional resistance training or sequential aerobic–resistance training.

A further limitation and central experimental challenge is the potential confounding effect of exercise dose. Because RT-AR replaces passive recovery with aerobic activity, comparisons with resistance training alone inherently differ in total movement, aerobic exposure, cumulative cardiovascular load, and likely energy expenditure. Such comparisons can establish the effectiveness of the overall RT-AR strategy but cannot determine whether any benefit is attributable specifically to the inter-set architecture. This distinction requires dose-matched sequential combined-training comparators, as discussed in Section 6.2.

A further unresolved issue is whether aerobic active recovery compromises resistance-training performance. Acute evidence is heterogeneous and derives predominantly from young or trained populations. It therefore remains unknown whether adults with obesity or metabolic syndrome can maintain the intended resistance-training volume, movement quality, and neuromuscular performance when passive recovery is replaced by aerobic activity. Any metabolic benefit of RT-AR should consequently be interpreted together with its effect on the resistance-training stimulus actually delivered, as discussed in Section 6.3.

Additional limitations include heterogeneity in populations, intervention type and duration, and biological material analyzed. Multi-omics outcomes are particularly sensitive to non-training exposures; co-occurring changes in microbiota, metabolites, adipokines, inflammatory markers, and DNA methylation do not by themselves establish causality. Future RT-AR trials should therefore characterize dietary intake, including total energy, fiber and protein; recent antibiotic exposure; probiotic/prebiotic use; smoking; alcohol intake; sleep; and habitual physical activity. Medication exposure should be documented prospectively, including metformin, GLP-1 receptor agonists, SGLT2 inhibitors, statins, and antihypertensive therapy. Drug name/class, dose, initiation or discontinuation, dose changes, and adherence/consistency of use should be recorded throughout the intervention, with stable regimens maintained where clinically appropriate and without interfering with necessary medical care. GLP-1 receptor agonists deserve particular attention because their growing use in obesity care can substantially alter the metabolic phenotype and may also modify gut microbiota composition, creating a major source of confounding in microbiome and metabolome analyses [98].

Sex should also be treated as a potential effect modifier rather than a background characteristic. A substantial proportion of the adipokine evidence summarized in this review derives from men, limiting direct generalization to women. Future RT-AR trials should ideally include both sexes, prespecify whether sex-stratified analyses are planned, and record menstrual or menopausal status where applicable. This is particularly important in multi-omics responder analyses, in which sex-related differences in body-fat distribution, hormonal milieu, inflammatory signaling, and metabolic adaptation could contribute to apparent inter-individual variability.

Cardiovascular safety should be addressed through individualized preparticipation assessment rather than routine testing based solely on obesity or metabolic syndrome. Obesity or MetS alone should not be interpreted as an indication for mandatory medical clearance or exercise stress testing. Assessment should consider current physical-activity status, known cardiovascular, metabolic or renal disease, signs or symptoms suggestive of cardiovascular disease, relevant comorbidities and medications, and planned exercise intensity, in accordance with contemporary clinical exercise-prescription guidance [9,10,90]. Exercise testing may be appropriate when clinically indicated—for example, in symptomatic individuals, selected patients with known cardiovascular disease, or when test results are expected to alter exercise prescription or supervision. RT-AR should therefore be regarded as a model requiring further validation in terms of efficacy, safety, and underlying mechanisms.

## 8. Conclusions

Established evidence supports aerobic, resistance, and combined training for improving body composition, cardiometabolic risk, physical fitness, and muscle function in adults with obesity and MetS. RT-AR reorganizes these established components by placing controlled low-impact aerobic activity within resistance-training inter-set recovery. Acute active-recovery studies demonstrate that this architecture is feasible in other populations, but no randomized chronic intervention study meeting the operational RT-AR definition was identified in adults with obesity or MetS. RT-AR should therefore be regarded as a testable training architecture rather than an established therapeutic modality.

The main unresolved question is whether embedding aerobic activity between resistance sets provides value beyond simply increasing total exercise dose. Any potential metabolic advantage must also be weighed against the possibility that active recovery reduces repetitions, volume-load, movement velocity, technical quality, or long-term strength and lean-mass adaptation. Likewise, microbiota, metabolomic, adipokine, and DNA-methylation pathways discussed in this review remain indirect, hypothesis-generating targets rather than established mechanisms of RT-AR.

Future trials should compare RT-AR with resistance training using passive recovery and with dose-matched sequential combined training, while quantifying both aerobic exposure and the resistance stimulus actually delivered. Clinical outcomes, adherence, body composition, strength, and resistance-set performance should be assessed together with carefully characterized diet, medication use, habitual physical activity, and sex-related factors. Multi-omics integration may then be used exploratorily to identify mediators, effect modifiers, and responder phenotypes [99,100], with metabolomic findings from conventional combined training providing a relevant indirect model for this approach [101]. The central research task is therefore to test whether RT-AR can increase supervised within-session movement while preserving the intended resistance-training stimulus and producing clinically meaningful effects independent of exercise-dose differences.

## Figures and Tables

**Table 1:** Hierarchy and directness of evidence informing the RT-AR hypothesis.

Evidence Level	Evidence Base	Main Domains/Outcomes	Relationship to RT-AR	Interpretation
Level 1. Direct chronic evidence for RT-AR	None identified in the dedicated PubMed/MEDLINE and Scopus searches in adults with overweight, obesity, abdominal obesity, or metabolic syndrome.	No chronic RT-AR clinical, metabolic, or multi-omics outcomes are currently available in the target population.	Would directly test the operational RT-AR model.	Evidence gap. Acute inter-set aerobic active-recovery studies in other populations provide proof-of-concept for the session architecture but do not establish chronic clinical efficacy.
Level 2. Indirect evidence from conventional exercise interventions	Aerobic training; resistance training; and sequential or concurrent combined aerobic-resistance training.	Body composition and central adiposity; glucose-insulin regulation; blood pressure; lipid profile; cardiorespiratory fitness; muscle strength; inflammation; circulating adipokines.	Supports the clinical and biological rationale for combining aerobic and resistance stimuli.	These data support the components from which RT-AR is constructed but cannot establish that embedding aerobic activity within inter-set recovery is superior to conventional training architectures.
Level 3. Mechanistic evidence informing the RT-AR hypothesis	Exercise studies examining gut microbiota, microbial metabolites, intestinal barrier function, adipokines/myokines, DNA methylation, gene expression, metabolomics, and related molecular pathways.	Microbiota and metabolites; SCFAs and bile acids; inflammatory and adipokine signaling; skeletal-muscle and adipose-tissue methylation; gene expression; multi-omics response patterns.	Provides candidate mechanisms, mediators, effect modifiers, and biomarkers for future RT-AR trials.	Indirect and hypothesis-generating. No RT-AR-specific microbiota, metabolomic, adipokine, or DNA-methylation signature has been established.

Note: The three levels indicate the directness of evidence in relation to the RT-AR hypothesis, not methodological quality or certainty of evidence. They do not constitute a GRADE or other formal evidence-quality assessment. Level 1 refers specifically to chronic intervention evidence meeting the operational RT-AR definition in adults with overweight, obesity, abdominal obesity, or metabolic syndrome. Acute inter-set active-recovery studies in other populations are considered proof-of-concept for feasibility of the session architecture and are discussed separately.

**Table 2:** Conceptual principles and illustrative test parameters for future experimental validation of RT-AR.

Protocol Component	General Principle Supported by Established Exercise Practice	Illustrative Author-Proposed Test Parameter	Monitoring/Criterion for Modification
Session frequency and structure	Supervised, progressive resistance training with low-impact aerobic activity embedded within inter-set recovery.	3 sessions/week, approximately 60 min/session. Week 1: full-body adaptation. Week 2: begin transition to an A/B organization at reduced volume. From week 3: full planned A/B volume, if tolerance and technique criteria are met. Each session begins with ~5 min of low-intensity aerobic warm-up and then follows the sequence resistance set → active recovery → next resistance set.	Record attendance, session duration, symptoms and completed work. Reduce total exposure if recovery between sessions is inadequate.
Resistance exercise selection	Use technically manageable exercises that cover major movement patterns and can be individualized to functional capacity.	Approximately 3–4 main exercises per session. Week 1 may use full-body organization; the A/B structure can begin in week 2 and reach full planned volume from week 3. Exercise variants remain individualized rather than fixed by BMI.	Movement quality, pain, range of motion, balance and ability to maintain planned technique. Substitute the exercise if these criteria are not met.
Resistance intensity and volume	Progressive, submaximal loading while avoiding routine training to failure.	Initial example: ~3–4 sets of 10–12 repetitions at approximately 60–70% 1RM or a load that permits ~2–3 RIR; later progression toward ~1–2 RIR if well tolerated.	RIR, set/session RPE, repetitions completed, external load and technical quality; where feasible, record repetition velocity or power. Reduce load or sets if planned RIR cannot be maintained or technique deteriorates.
Movement control and breathing	Controlled repetitions with stable technique and avoidance of unnecessary prolonged Valsalva maneuvers.	Illustrative tempo: approximately 2–3 s eccentric and 1–2 s concentric.	Modify load, range of motion or tempo if breathing becomes uncontrolled or technique cannot be reproduced across sets.
Active-recovery modality	Low-impact aerobic activity should minimize orthopedic burden and not become an additional high-intensity interval.	Treadmill walking, stationary cycling or elliptical exercise; modality selected according to mobility, balance and orthopedic tolerance.	Pain, gait/balance quality and technical readiness for the subsequent resistance set. Change modality when needed.
Active-recovery intensity	Low-to-moderate intensity that maintains movement without materially impairing the next resistance set.	Illustrative starting range: ~50–60% HRmax and approximately RPE 2–4/10, with conversational breathing maintained.	HR, RPE, talk test/breathing, symptoms, repetitions completed, external load and planned RIR in the subsequent set; where feasible, monitor repetition velocity or power. Reduce active-recovery intensity or duration if repeated-set performance deteriorates beyond the prespecified tolerance.
Active-recovery duration	Replace part of passive inter-set recovery while preserving resistance-training quality.	Approximately 1–2 min after each resistance set; shorter bouts may be used during initial adaptation.	Shorten the bout or introduce passive recovery if technique, planned RIR or cardiorespiratory tolerance deteriorates.
Progression	Progress only after the current workload is tolerated with stable technique and the planned resistance-training dose is maintained.	Illustrative rule: after successful completion of the planned repetitions at target RIR with stable technique across two consecutive sessions, increase resistance load by a small increment (e.g., ~2–5%); progress active-recovery duration toward 2 min before increasing intensity.	Progress active-recovery exposure only when planned resistance volume and technique are maintained. Do not progress when pain, excessive fatigue, disproportionate dyspnea, abnormal physiological responses or technique deterioration is present.
Modification/termination	Dose should be individualized and reduced when safety or technical criteria are not met.	Possible modifications: reduce resistance load/sets, increase RIR, shorten or slow active recovery, change aerobic modality, or temporarily restore passive recovery.	Terminate the session for chest pain, presyncope/syncope, severe or disproportionate dyspnea, acute neurologic symptoms, acute musculoskeletal pain, or clinically concerning cardiovascular responses; reassess before resuming.

Note: Values in the “Illustrative author-proposed test parameter” column are candidate settings for prospective validation. They are provided to make the experimental framework reproducible and should not be interpreted as optimal or recommended clinical RT-AR parameters. HRmax, age-predicted maximal heart rate; RIR, repetitions in reserve; RPE, rating of perceived exertion.

**Table 3:** General principles for individualizing exercise selection within the proposed RT-AR framework.

Clinical/Functional Consideration	General Individualization Principle	Examples of Possible Modification
Reduced mobility or balance	Reduce technical complexity and increase external support when this improves stability and confidence.	Supported squat or sit-to-stand; rails/TRX when appropriate; stationary cycling instead of treadmill walking when balance is limiting.
Low back symptoms or limited lumbopelvic control	Minimize poorly tolerated spinal loading and prioritize positions that can be maintained without compensatory trunk motion.	Supported rowing; reduced hip-hinge range; lower external load; alternative machine/cable variants.
Low relative upper-body strength	Scale relative load while preserving the intended movement pattern.	Elevated push-up; machine or cable press/pull; adjusted support height or external resistance.
Abdominal-wall control limitations	Avoid exercise variants that provoke uncontrolled abdominal bulging, excessive straining or loss of trunk control.	Lower load or range of motion; isometric or anti-extension trunk-control variants; alternative supported positions.
Reduced cardiorespiratory tolerance	Maintain active recovery below the level that compromises technique or the subsequent resistance set.	Slower walking/cycling; shorter active-recovery bout; lower-resistance modality; temporary passive recovery.
Technique deterioration, pain or excessive fatigue	Modify the dose rather than progressing automatically.	Reduce resistance load or number of sets, increase RIR, change exercise variant, shorten active recovery, or extend passive transition time.

Note: The examples illustrate possible adaptations and are not BMI-specific or diagnosis-specific exercise prescriptions. Exercise selection should be individualized according to functional assessment, symptoms, orthopedic tolerance, and technical competence.

## Data Availability

No new data were created or analyzed in this study. Data sharing is not applicable to this article.

## Supplementary Materials

The following supporting information can be downloaded at https://www.mdpi.com/article/10.3390/jcm15176671/s1, Table S1: Database-specific electronic search strategies, search dates, limits, and record yields; Table S2: Eligibility criteria and evidence-selection framework; Table S3: Screening flow for the dedicated RT-AR search in adults with overweight, obesity, or metabolic syndrome.

## Abbreviations

The following abbreviations are used in this manuscript:

RT-AR	Resistance training with aerobic active recovery
MetS	Metabolic syndrome
BMI	Body mass index
SCFA	Short-chain fatty acids
LPS	Lipopolysaccharide
FXR	Farnesoid X receptor
TGR5	G protein-coupled bile acid receptor 1
AhR	Aryl hydrocarbon receptor
HDL-C	High-density lipoprotein cholesterol
LDL-C	Low-density lipoprotein cholesterol
HOMA-IR	Homeostatic Model Assessment for Insulin Resistance
QUICKI	Quantitative Insulin Sensitivity Check Index
RPE	Rating of perceived exertion
DRA	Diastasis recti abdominis
DNAm	DNA methylation
