# Supplementary material for: Resistance Training with Aerobic Active Recovery in Obesity and Metabolic Syndrome: Translational Rationale, Proposed Training Protocol, and Multi-Omics Research Framework—A Narrative Review
Source: J Clin Med. 2026 Aug 28;15(17):6671. doi: 10.3390/jcm15176671 (PMC13566755; doi:10.3390/jcm15176671)
Supplement: Supplementary file 1 [file jcm-15-06671-s001.zip › jcm-4532152-supplementary.pdf]

Supplementary Materials

Resistance Training with Aerobic Active Recovery in Patients with Obesity and Metabolic Syndrome: Multi-Omics Perspectives on the Interplay Between Gut Microbiota, DNA Methylation, and Adipokine Regulation—A Narrative Review

Contents

- Supplementary Methods: Literature-search framework and study-selection approach.
- Table S1: Database-specific electronic search strategies, search dates, limits, and record yields.
- Table S2: Eligibility criteria and evidence-selection framework.
- Table S3: Screening flow for the dedicated RT-AR search in adults with overweight, obesity, or metabolic syndrome.

Supplementary Methods

The supplementary searches were organized in layers consistent with the narrative, hypothesis-generating purpose of the review. PubMed/MEDLINE was the original biomedical database because it provides controlled Medical Subject Headings (MeSH) and broad coverage of clinical and mechanistic literature. Scopus was added during revision to broaden cross-disciplinary coverage and to independently verify the central research-gap statement concerning resistance training with aerobic active recovery (RT-AR). Broad evidence-mapping searches were used to identify higher-level evidence on exercise in obesity and metabolic syndrome; these searches were not treated as a pooled systematic-review screening cohort. Dedicated high-sensitivity searches were used to determine whether aerobic activity performed during inter-set recovery had been directly evaluated, first without population restriction and then specifically in adults with overweight, obesity, abdominal obesity, or metabolic syndrome. Additional domain-specific PubMed/MEDLINE searches mapped evidence concerning gut microbiota and DNA methylation/epigenetic regulation. Exact database syntax, search dates, limits, and record yields are reported in Table S1.

For the dedicated target-population RT-AR search, duplicate records were identified by DOI and normalized-title matching. Titles and abstracts of all unique records were screened against the prespecified criteria in Table S2. Full text was assessed when the structure of the recovery period could not be determined reliably from the title and abstract. Screening was performed by K.M. and the eligibility decisions were subsequently verified by A.S.; uncertainties were resolved by discussion and consensus. The screening flow is summarized in Table S3. Because the article is a narrative review rather than a systematic review of RT-AR efficacy, no PRISMA flow diagram or formal risk-of-bias assessment was applied to the broad evidence-mapping searches.

Table S1. Database-specific electronic search strategies, search dates, limits, and record yields.

ID	Database / purpose	Exact search string / limits	Date	Records
A1	PubMed/MEDLINE – broad exercise evidence mapping	("Exercise"[MeSH Terms] OR "Exercise Therapy"[MeSH Terms] OR "Resistance Training"[MeSH Terms] OR "High-Intensity Interval Training"[MeSH Terms] OR "Physical Conditioning, Human"[MeSH Terms] OR exercise[Title] OR exercises[Title] OR training[Title] OR "physical activity"[Title] OR "resistance training"[Title] OR "aerobic training"[Title] OR "combined training"[Title] OR "concurrent training"[Title]) AND ("Obesity"[MeSH Terms] OR obesity[Title] OR obese[Title] OR "Metabolic Syndrome X"[MeSH Terms] OR "metabolic syndrome"[Title]) NOT ("Child"[MeSH Terms] OR "Adolescent"[MeSH Terms]) AND (systematicreview[Filter] OR meta-analysis[Publication Type]). Limits: preceding 10 years; systematic review/meta-analysis.	11 Aug 2026	696
A2	Scopus – broad exercise evidence mapping	TITLE(exercise OR exercises OR training OR "physical activity" OR "resistance training" OR "aerobic training" OR "combined training" OR "concurrent training") AND TITLE(obesity OR obese OR "metabolic syndrome") AND TITLE-ABS-KEY("systematic review" OR "systematic reviews" OR "meta-analysis" OR "meta analysis") AND NOT TITLE(child OR children OR adolescent OR adolescents) AND PUBYEAR > 2015	20 Aug 2026	586
B1	PubMed/MEDLINE – direct RT-AR, unrestricted population	("active recovery"[Title/Abstract] OR "active rest"[Title/Abstract] OR "aerobic recovery"[Title/Abstract] OR (inter-set[Title/Abstract] AND recovery[Title/Abstract]) OR (interset[Title/Abstract] AND recovery[Title/Abstract]) OR ("between sets"[Title/Abstract] AND recovery[Title/Abstract])) AND ("Resistance Training"[MeSH Terms] OR "resistance training"[Title/Abstract] OR "resistance exercise"[Title/Abstract] OR "strength training"[Title/Abstract] OR "weight training"[Title/Abstract])	20 Aug 2026	270
B2	Scopus – direct RT-AR, unrestricted population	TITLE-ABS-KEY("active recovery" OR "active rest" OR "active inter-set recovery" OR "active interset recovery" OR "inter-set active recovery" OR "interset active recovery" OR "between-set active recovery" OR "between set active recovery" OR "inter-set recovery" OR "interset recovery" OR "between-set recovery") AND TITLE-ABS-KEY("resistance training" OR "resistance exercise" OR "strength training" OR "weight training")	20 Aug 2026	158
B3	PubMed/MEDLINE – direct RT-AR + overweight/obesity/MetS	("active recovery"[Title/Abstract] OR "active rest"[Title/Abstract] OR (active[Title/Abstract] AND inter-set[Title/Abstract] AND recovery[Title/Abstract]) OR (active[Title/Abstract] AND interset[Title/Abstract] AND recovery[Title/Abstract]) OR (inter-set[Title/Abstract] AND recovery[Title/Abstract]) OR (interset[Title/Abstract] AND recovery[Title/Abstract]) OR ("between sets"[Title/Abstract] AND recovery[Title/Abstract]) OR "between-set recovery"[Title/Abstract]) AND ("Resistance Training"[MeSH Terms] OR "resistance training"[Title/Abstract] OR "resistance exercise"[Title/Abstract] OR "strength training"[Title/Abstract] OR "weight training"[Title/Abstract]) AND ("Obesity"[MeSH Terms] OR obesity[Title/Abstract] OR obese[Title/Abstract] OR overweight[Title/Abstract] OR "abdominal obesity"[Title/Abstract] OR "Metabolic Syndrome"[MeSH Terms] OR "metabolic syndrome"[Title/Abstract])	20 Aug 2026	14
B4	Scopus – direct RT-AR + overweight/obesity/MetS	TITLE-ABS-KEY("active recovery" OR "active rest" OR "active inter-set recovery" OR "active interset recovery" OR "inter-set active recovery" OR "interset active recovery" OR "between-set active recovery" OR "between set active recovery" OR "inter-set recovery" OR "interset recovery" OR "between-set recovery") AND TITLE-ABS-KEY("resistance training" OR "resistance exercise" OR "strength training" OR "weight training") AND TITLE-ABS-KEY(obesity OR obese OR overweight OR "abdominal obesity" OR "metabolic syndrome")	20 Aug 2026	17
C1	PubMed/MEDLINE – microbiota, broad	("Exercise"[MeSH Terms] OR "Resistance Training"[MeSH Terms] OR exercise[Title/Abstract] OR "aerobic training"[Title/Abstract] OR "resistance training"[Title/Abstract] OR "combined training"[Title/Abstract] OR "concurrent training"[Title/Abstract]) AND ("Gastrointestinal Microbiome"[MeSH Terms] OR "gut microbiota"[Title/Abstract] OR "gut microbiome"[Title/Abstract] OR microbiota[Title/Abstract] OR microbiome[Title/Abstract]) AND ("Obesity"[MeSH Terms] OR obesity[Title/Abstract] OR obese[Title/Abstract] OR overweight[Title/Abstract] OR "Metabolic Syndrome"[MeSH Terms] OR "metabolic syndrome"[Title/Abstract])	20 Aug 2026	541
C2	PubMed/MEDLINE – microbiota, systematic reviews/meta-analyses	("Exercise"[MeSH Terms] OR "Resistance Training"[MeSH Terms] OR exercise[Title/Abstract] OR "aerobic training"[Title/Abstract] OR "resistance training"[Title/Abstract] OR "combined training"[Title/Abstract] OR "concurrent training"[Title/Abstract]) AND ("Gastrointestinal Microbiome"[MeSH Terms] OR "gut microbiota"[Title/Abstract] OR "gut microbiome"[Title/Abstract] OR microbiota[Title/Abstract] OR microbiome[Title/Abstract]) AND ("Obesity"[MeSH Terms] OR obesity[Title/Abstract] OR obese[Title/Abstract] OR overweight[Title/Abstract] OR "Metabolic Syndrome"[MeSH Terms] OR "metabolic syndrome"[Title/Abstract]) AND (systematicreview[Filter] OR meta-analysis[Publication Type])	20 Aug 2026	18

ID	Database / purpose	Exact search string / limits	Date	Records
C3	PubMed/MEDLINE – microbiota, intervention/mechanistic layer	("Exercise"[MeSH Terms] OR "Resistance Training"[MeSH Terms] OR exercise[Title/Abstract] OR "aerobic training"[Title/Abstract] OR "resistance training"[Title/Abstract] OR "combined training"[Title/Abstract] OR "concurrent training"[Title/Abstract]) AND ("Gastrointestinal Microbiome"[MeSH Terms] OR "gut microbiota"[Title/Abstract] OR "gut microbiome"[Title/Abstract] OR microbiota[Title/Abstract] OR microbiome[Title/Abstract]) AND ("Obesity"[MeSH Terms] OR obesity[Title/Abstract] OR obese[Title/Abstract] OR overweight[Title/Abstract] OR "Metabolic Syndrome"[MeSH Terms] OR "metabolic syndrome"[Title/Abstract]) AND (randomized controlled trial[Publication Type] OR clinical trial[Publication Type] OR intervention[Title/Abstract] OR training[Title/Abstract])	20 Aug 2026	197
C4	PubMed/MEDLINE – microbiota, clinical/intervention trials	("Exercise"[MeSH Terms] OR "Exercise Therapy"[MeSH Terms] OR "Resistance Training"[MeSH Terms] OR exercise[Title/Abstract] OR "aerobic training"[Title/Abstract] OR "resistance training"[Title/Abstract] OR "combined training"[Title/Abstract] OR "concurrent training"[Title/Abstract]) AND ("Gastrointestinal Microbiome"[MeSH Terms] OR "gut microbiota"[Title/Abstract] OR "gut microbiome"[Title/Abstract] OR microbiota[Title/Abstract] OR microbiome[Title/Abstract]) AND ("Obesity"[MeSH Terms] OR obesity[Title/Abstract] OR obese[Title/Abstract] OR overweight[Title/Abstract] OR "Metabolic Syndrome"[MeSH Terms] OR "metabolic syndrome"[Title/Abstract]) AND (randomized controlled trial[Publication Type] OR controlled clinical trial[Publication Type] OR clinical trial[Publication Type])	20 Aug 2026	43
D1	PubMed/MEDLINE – DNA methylation/epigenome, broad	("Exercise"[MeSH Terms] OR "Exercise Therapy"[MeSH Terms] OR "Resistance Training"[MeSH Terms] OR exercise[Title/Abstract] OR "aerobic training"[Title/Abstract] OR "resistance training"[Title/Abstract] OR "combined training"[Title/Abstract] OR "concurrent training"[Title/Abstract]) AND ("DNA Methylation"[MeSH Terms] OR "DNA methylation"[Title/Abstract] OR methylation[Title/Abstract] OR methylome[Title/Abstract] OR epigenome[Title/Abstract] OR epigenetic[Title/Abstract] OR "epigenetic age"[Title/Abstract]) AND ("Obesity"[MeSH Terms] OR obesity[Title/Abstract] OR obese[Title/Abstract] OR overweight[Title/Abstract] OR "Metabolic Syndrome"[MeSH Terms] OR "metabolic syndrome"[Title/Abstract])	20 Aug 2026	250
D2	PubMed/MEDLINE – DNA methylation/epigenome, systematic reviews/meta-analyses	("Exercise"[MeSH Terms] OR "Exercise Therapy"[MeSH Terms] OR "Resistance Training"[MeSH Terms] OR exercise[Title/Abstract] OR "aerobic training"[Title/Abstract] OR "resistance training"[Title/Abstract] OR "combined training"[Title/Abstract] OR "concurrent training"[Title/Abstract]) AND ("DNA Methylation"[MeSH Terms] OR "DNA methylation"[Title/Abstract] OR methylation[Title/Abstract] OR methylome[Title/Abstract] OR epigenome[Title/Abstract] OR epigenetic[Title/Abstract] OR "epigenetic age"[Title/Abstract]) AND ("Obesity"[MeSH Terms] OR obesity[Title/Abstract] OR obese[Title/Abstract] OR overweight[Title/Abstract] OR "Metabolic Syndrome"[MeSH Terms] OR "metabolic syndrome"[Title/Abstract]) AND (systematicreview[Filter] OR meta-analysis[Publication Type])	20 Aug 2026	6
D3	PubMed/MEDLINE – DNA methylation/epigenome, clinical/intervention trials	("Exercise"[MeSH Terms] OR "Exercise Therapy"[MeSH Terms] OR "Resistance Training"[MeSH Terms] OR exercise[Title/Abstract] OR "aerobic training"[Title/Abstract] OR "resistance training"[Title/Abstract] OR "combined training"[Title/Abstract] OR "concurrent training"[Title/Abstract]) AND ("DNA Methylation"[MeSH Terms] OR "DNA methylation"[Title/Abstract] OR methylation[Title/Abstract] OR methylome[Title/Abstract] OR epigenome[Title/Abstract] OR epigenetic[Title/Abstract] OR "epigenetic age"[Title/Abstract]) AND ("Obesity"[MeSH Terms] OR obesity[Title/Abstract] OR obese[Title/Abstract] OR overweight[Title/Abstract] OR "Metabolic Syndrome"[MeSH Terms] OR "metabolic syndrome"[Title/Abstract]) AND (randomized controlled trial[Publication Type] OR controlled clinical trial[Publication Type] OR clinical trial[Publication Type])	20 Aug 2026	15
D4	PubMed/MEDLINE – ADIPOQ/LEP + methylation + exercise	(ADIPOQ[Title/Abstract] OR adiponectin[Title/Abstract] OR LEP[Title/Abstract] OR leptin[Title/Abstract]) AND ("DNA methylation"[Title/Abstract] OR methylation[Title/Abstract] OR methylome[Title/Abstract] OR epigenetic[Title/Abstract]) AND (exercise[Title/Abstract] OR training[Title/Abstract] OR "physical activity"[Title/Abstract] OR "resistance training"[Title/Abstract] OR "aerobic training"[Title/Abstract] OR "combined training"[Title/Abstract])	20 Aug 2026	33
D5	PubMed/MEDLINE – ADIPOQ/LEP + methylation + exercise + obesity/MetS	(ADIPOQ[Title/Abstract] OR adiponectin[Title/Abstract] OR LEP[Title/Abstract] OR leptin[Title/Abstract]) AND ("DNA methylation"[Title/Abstract] OR methylation[Title/Abstract] OR methylome[Title/Abstract] OR epigenetic[Title/Abstract]) AND (exercise[Title/Abstract] OR training[Title/Abstract] OR "physical activity"[Title/Abstract] OR "resistance training"[Title/Abstract] OR "aerobic training"[Title/Abstract] OR "combined training"[Title/Abstract]) AND (obesity[Title/Abstract] OR obese[Title/Abstract] OR overweight[Title/Abstract] OR "abdominal obesity"[Title/Abstract] OR "metabolic syndrome"[Title/Abstract])	20 Aug 2026	23

Note: RT-AR, resistance training with aerobic active recovery; MetS, metabolic syndrome. PubMed/MEDLINE may issue phrase-index warnings for uncommon hyphenated expressions. In the final target-population query, unsupported exact phrases were therefore represented using Title/Abstract word combinations. Scopus syntax was adapted to TITLE, TITLE-ABS-KEY, and PUBYEAR fields.

Table S2. Eligibility criteria and evidence-selection framework.

Element	Inclusion / prioritization	Exclusion / qualification
Population	For the dedicated target-population RT-AR search: adults with overweight, obesity, abdominal obesity, or metabolic syndrome. The unrestricted direct RT-AR search had no population restriction and was used to identify proof-of-concept studies and terminology.	Pediatric populations excluded from the target clinical evidence set. Non-human studies excluded as direct clinical evidence.
Intervention	Resistance exercise in which a planned aerobic activity (e.g., treadmill walking/running, stationary cycling, elliptical or comparable low-impact aerobic modality) is performed during the inter-set recovery period before the next resistance set.	Active rest consisting only of low-load resistance repetitions or stretching; aerobic activity only between HIIT/aerobic intervals; aerobic and resistance components performed as separate sequential blocks; circuit/HIFT without explicit aerobic inter-set recovery.
Study design	Primary human intervention studies. Acute studies were retained as proof-of-concept for the session architecture; chronic/repeated training studies were required for the main clinical research-gap claim.	Reviews, editorials, commentaries, and protocols without relevant intervention data excluded from the direct-evidence set.
Narrative evidence hierarchy	Systematic reviews/meta-analyses first; then randomized or controlled intervention studies; then mechanistic primary studies, with priority to adults with obesity/MetS and to interventions most comparable with combined training or RT-AR.	Broad search results were not treated as a systematic-review screening cohort. Preclinical/cellular studies were used only to support specific mechanisms, not clinical efficacy.
Screening and disagreements	Dedicated target-population RT-AR search screened by K.M. and verified by A.S.; uncertainty resolved by discussion and consensus. Full text checked when intervention architecture was unclear from title/abstract.	Formal dual-independent PRISMA screening was not applied to the broad narrative evidence-mapping searches.
Duplicates / supplementary searching	Duplicates in the dedicated PubMed + Scopus target search removed by DOI and normalized-title matching. Reference lists of key reviews/directly relevant studies checked manually.	No formal citation-network analysis was used.

The dedicated direct-evidence definition required planned aerobic activity to occur during the recovery interval between resistance-training sets. Conventional combined training, interval resistance training using low-load resistance repetitions as active rest, and aerobic active recovery occurring only between HIIT intervals were not classified as RT-AR.

Table S3. Screening flow for the dedicated RT-AR search in adults with overweight, obesity, or metabolic syndrome.

Screening stage	n	Details
Records identified in PubMed/MEDLINE	14	Dedicated target-population RT-AR search; no date restriction.
Records identified in Scopus	17	Dedicated target-population RT-AR search; no date restriction.
Total records before deduplication	31	PubMed/MEDLINE + Scopus.
Duplicates removed	11	Matched by DOI and normalized title.
Unique records screened by title/abstract	20	All unique records from the dedicated target-population search.
Records excluded after title/abstract screening	17	Clearly ineligible intervention architecture, population, study type, or recovery context.
Full-text reports assessed for eligibility	3	Full text checked because the abstract did not allow reliable determination of how active recovery was integrated into the resistance session.

Screening stage	n	Details
Full-text reports excluded	3	Two used low-load resistance repetitions as active rest and placed aerobic exercise in a separate sequential block; one used stretching between resistance stations and aerobic active recovery only within a subsequent aerobic block.
Chronic RT-AR intervention studies meeting the target-population definition	0	No study used planned aerobic activity as the inter-set recovery between resistance sets in adults with overweight, obesity, abdominal obesity, or metabolic syndrome.

Interpretation: The unrestricted direct RT-AR searches identified acute inter-set active-recovery experiments in non-clinical populations, supporting proof-of-concept for the session architecture. The zero count in Table S3 refers specifically to chronic interventions meeting the prespecified target-population and RT-AR architecture criteria.
